# Clinically significant germline pathogenic variants are missed by tumor genomic sequencing

**DOI:** 10.1038/s41525-023-00374-9

**Published:** 2023-10-13

**Authors:** Leigh Anne Stout, Cynthia Hunter, Courtney Schroeder, Nawal Kassem, Bryan P. Schneider

**Affiliations:** 1 Indiana University School of Medicine, Indianapolis, IN USA; 2 Indiana University Health Precision Genomics, Indianapolis, IN USA

**Keywords:** Cancer genomics, Cancer genetics

## Abstract

A germline pathogenic variant may be present even if the results of tumor genomic sequencing do not suggest one. There are key differences in the assay design and reporting of variants between germline and somatic laboratories. When appropriate, both tests should be completed to aid in therapy decisions and determining optimal screening and risk-reduction interventions.

Identification of germline pathogenic variants (PV) for both patients with cancer and their unaffected relatives has major health implications. Germline PV carriers, and their family members, may benefit from increased screening and appropriate risk reduction interventions^1^. While clearly beneficial for family members who carry the PV but do not yet have cancer, this is also becoming increasingly relevant for the patient as tumor genomic sequencing is being implemented in earlier-stage cancers rather than only patients with heavily pre-treated metastatic disease^2^. Increasingly, germline PVs serve not only as a predictor of cancer risk but also may predict benefit to specific targeted agents such as PARP-inhibition for patients with specific tumors who harbor PVs in the homologous recombination genes and belzutifan for those with germline *VHL* PVs^3^. While some patients benefit from either somatic or germline PVs in candidate genes, some settings benefit only, or preferentially, with germline PVs. For example, advanced breast cancer patients with a germline *BRCA1* or *BRCA2* PV have a greater benefit to PARP inhibition than individuals with a somatic *BRCA1* or *BRCA2* PV, and the FDA-approval for PARP inhibition is currently confined to those with a germline PV^4^.

Tumor genomic sequencing, which has been integrated into routine oncology care for many cancer patients^5,6^, has also become an avenue for incidental germline PV discovery. While these tests are designed to uncover genetic PVs that may be driving tumor growth with the goal of identifying additional treatment options to target those PVs, the PVs may be germline in origin or acquired somatically during carcinogenesis. Understanding the origin (somatic vs. germline) is critical in determining appropriate therapeutic options and providing appropriate follow-up for at-risk relatives. Importantly, pathogenic germline PVs are uncovered in ~11–16% of individuals undergoing tumor genomic sequencing^7–13^. While tumor genomic sequencing has demonstrated the ability to identify germline carriers, sometimes incidentally, it is not comprehensive and does not identify all pathogenic PVs originating from the germline^14^.

Many tumor-genomic sequencing platforms do not employ paired germline and thus inferences regarding the origin of a PV (somatic vs. germline) are frequently made based on several factors. These include the specific gene that is mutated, the patient’s personal/family history, and the variant allele frequency^15,16^. However, a somatic test report that does not reveal a PV suspected of being germline in origin does not exclude the existence of one. Prior studies have found between 8% and 18% of germline PVs were missed by tumor genomic sequencing^17,18^. The inability to perfectly uncover all germline risk-conferring PVs is due to several key differences between somatic and germline testing including assay differences, bioinformatic processing, and differences in interpretation of pathogenicity of the same variant between somatic and germline labs. Even among somatic labs, there are notable differences in variant reporting and assay design. Of those that have a paired-normal sample, some labs will filter germline variants off the report. Therefore, the results of tumor genomic sequencing cannot be used as a surrogate for germline testing. We report on the experience of identifying germline PVs that were missed by tumor genomic sequencing through the Indiana University (IU) Health Precision Genomics Program.

## Methods

### Results review

The methods of this study were carried out in accordance with relevant guidelines and regulations approved by the Indiana University Health IRB. The study received a waiver of consent based on federal regulation 45 CFR 164.612(i).

Patients seen through the IU Health Precision Genomics program have all had somatic tumor genomic sequencing completed on formalin-fixed paraffin-embedded (FFPE) specimens through a CLIA-certified lab that reports an average sequencing read depth of at least ×440. Genomic data from the somatic lab are securely transferred into the LifeOmic Platform by the sequencing lab which then triggers pre-configured ingestion workflows customized to the source formatting. The VCF and BAM files provided by the sequencing lab are loaded into the LifeOmic Platform to allow for a more comprehensive review of all identified variants, regardless of inclusion on the final clinical pdf report. By default, only variants that pass the originating sequencing lab’s quality control metrics in the VCF are ingested into the LifeOmic database. The LifeOmic Platform is a commercially available precision health platform licensed by IU Health that provides an interactive user interface to easily filter and review all sequencing variants in a VCF file. Somatic data were reviewed in the LifeOmic Platform by a licensed genetic counselor.

When clinically indicated based on personal/family history or findings on tumor sequencing, germline testing was also completed at a commercially available CLIA-certified lab. This testing was either done by another provider prior to their visit to the IU Health Precision Genomics program or as part of their workup through the program.

A list of known germline PVs that were not detected or not determined to be pathogenic by the somatic lab was compiled. Genes were only included if they were analyzed by both the somatic and germline platforms. A licensed genetic counselor consulted with the somatic lab for clarity when a germline PV was missed by their test.

If a variant was classified as pathogenic or likely pathogenic by a germline lab but was classified as either a VUS, likely benign, or benign by the somatic lab it was labeled as a difference in pathogenicity. If a germline PV was not in the somatic report and there was no sequencing coverage of that genomic position in the raw mapping file (BAM), it was labeled as a difference in assay design, which indicates the assay was not capable of detecting that aberration. If a germline PV was present (>1 variant read) in the somatic raw mapping file (BAM) (confirming the variant was detectable by the sequencing assay) but not listed on the somatic report, it was labeled as a bioinformatic filtering issue confirming that the omission was subsequent to its detection.

Between April 18, 2014, and May 11, 2023, there have been 7627 patients who have had somatic tumor genomic sequencing by our program. Within the 7627 patients, there have been 320 germline PVs confirmed by a CLIA-validated germline test. Of those, 21 PVs (6.6%) were not detected by the somatic testing lab. The reasons for these PVs being missed by the somatic lab(s) are summarized in Table 1.

**Table 1:** Description of germline pathogenic variants that were missed by somatic testing in patients with advanced cancer.

	Age at diagnosis, race, sex	Tumor sequenced	Other pertinent medical history	Gene	PV	Germline lab	Somatic test (lab)
*Assay differences* (*n* = 13)							
Patient 1	44, AAF	Breast	Papillary thyroid cancer dx 42	*BRCA1*	Del exon 23	Invitae	MI Tumor Seek Hybrid (Caris)
Patient 2	62, WM	Prostate	–	*CDKN2A*	c.9_32dup24	Invitae	FoundationOne CDx (FMI)
Patient 3	35, WF	Breast	–	*CHEK2*	Ex8_9del^a^	Myriad	GEM ExTra (Ashion) and FoundationOne Liquid (FMI)
Patient 4	66, WM	Prostate	–	*CHEK2*	Ex8_9del^a^	Ambry	FoundationOne CDx (FMI)
Patient 5	41, WF	Colorectal	Appendiceal carcinoid dx 41	*CHEK2*	EX2_3′UTRdel	Ambry	GEM ExTra (Ashion)
Patient 6	29, unknown F	Colorectal	MSI-high tumor	*MLH1*	Ex5_6del	Ambry	GPS Cancer (NantOmics)and FoundationACT (FMI)
Patient 7	42, WM	Colorectal	MSI-high tumor	*MSH2*	5’UTR_EX3del	Ambry	GPS Cancer (NantOmics)and FoundationOne Heme (FMI)
Patient 8	72, WM	Colorectal	MSI-high tumor; ampullary cancer dx 53	*MSH2*	5’UTR_EX6del	Ambry	MI Tumor Seek Hybrid (Caris)
Patient 9	44, WF	Colorectal	MSI-high tumor; pleiomorphic sarcoma dx 43	*MSH2*	Del exons 1–6	Myriad	MI Tumor Seek Hybrid (Caris)
Patient 10	42, WM	Glioblastoma	MSI-high tumor	*MSH2*	Del exon 6	Prevention Genetics	MI Tumor Seek Hybrid (Caris)
Patient 11	66, WF	Mucosal melanoma	breast cancer dx 51; breast cancer dx 66	*PALB2*	EX11del^a^	Myriad	FoundationOne CDx (FMI)
Patient 12	52, WM	Colorectal	Prostate cancer dx 61; also carries germline *BRCA2* PV that was identified in tumor	*PALB2*	EX11del^a^	Ambry	GEM ExTra (Ashion) and FoundationOne Liquid (FMI)
Patient 13	46, WM	Colorectal	MS-stable tumor	*PMS2*	EX9_10del	Ambry	FoundationOne (FMI) and FoundationACT (FMI)
*Bioinformatic filtering* (*n* = 5)							
Patient 14	54, WM	Pancreatic	–	*ATM*	c.2251-10T > G	Ambry	FoundationOne CDx (FMI)
Patient 15	71, WM	Prostate	Melanoma dx 69	*MITF*	E318K^a^	Ambry	FoundationOne CDx (FMI)
Patient 16	51, WF	Leiomyosarcoma	Also carries germline *TP53* PV that was identified in tumor	*MITF*	E318K^a^	Ambry	FoundationOne Liquid CDx (FMI)
Patient 17	77, unknown M	Lung	MS-stable tumor	*PMS2*	c.1831dup	Invitae	MI Tumor Seek Hybrid (Caris)
Patient 18	48, WF	Ovarian	–	*TP53*	M246V	Ambry	GPS Cancer (NantOmics)
*Differences in pathogenicity* (*n* = 3)							
Patient 19	33, WF	Breast	DCIS dx 37	*CHEK2*	T367fs^a^15	Ambry	FoundationACT (FMI)
Patient 20	71, WF	Pancreatic	MSI status of pancreatic cancer could not be determined; Endometrial cancer dx 61 (MSI status unknown)	*MSH6*	L370S^a^	Invitae	FoundationOne CDx (FMI)
Patient 21	47, WM	Osteogenic sarcoma	MS-stable sarcoma; testicular cancer dx	*MSH6*	L370S^a^	Ambry	FoundationOne Heme (FMI)

*AA* African American, *W* White, *F* Female, *M* Male, *FMI* Foundation Medicine, Inc.

^a^Denotes this PV was identified in more than 1 patient.

The identification of risk-conferring alleles is common, and undercovering incidental germline findings (even when not expected based on personal and family history) is not rare. In clinical practice, NCCN guidelines recommend consideration of formal germline testing to verify the presence of a risk-conferring germline PV that is identified on a somatic tumor test. The relative overlap has the potential to falsely lead patients and providers that a negative tumor genomic sequence report can obviate the need to proceed with formal germline testing, even when risk is recommended.

There are multiple reasons a germline PV will not be listed on a somatic report or interpreted as pathogenic by a somatic laboratory. As highlighted in this report, these may include differences in the number of genes/variants tested (not considered here), in definitions of pathogenicity, in bioinformatic filtering, and in assay design.

With regard to differences in assay design, many commercially available somatic testing labs will not detect certain structural alterations, including small deletions and duplications. These types of PVs are equally impactful on gene function as other types of PVs. The likelihood of a deletion or duplication is gene-specific, with over a quarter of PVs in *MSH2* and *PMS2* the result of a deletion or duplication^19^. In our cohort, 13 of 21 germline PVs were missed due to assay differences between somatic and germline labs. Each of these 13 PVs (10 unique PVs) were in genes with high or moderate penetrance which would have significant implications for the patients and their relatives’ medical care. In some cases, these PVs may still be driving cancer growth and be a high-value drug target, including 3 of our patients who had either a germline *BRCA1* or *PALB2* PV.

Perhaps a more challenging explanation for germline PVs being missed by a somatic test is a function of bioinformatic processing by the somatic laboratory. In the case of the missed *PMS2* c.1831dup germline PV in this report, the somatic lab identified the PV but filtered it off the report because it fell within a region of homology with the *PMS2CL* pseudogene. Interestingly, this patient had a microsatellite stable tumor which is not uncommon in individuals with *PMS2* or *MSH6*-related Lynch syndrome^20^. In fact, microsatellite instability was not detected in any of our patients with germline *PMS2* or *MSH6* PVs. Therefore, relying on the microsatellite stability result is also an imperfect method of identifying individuals with Lynch syndrome. For the missed *ATM* germline PV in this report, the somatic lab detected this PV but was only identified by our group after reviewing the variants in the LifeOmic platform. It had been filtered off the somatic report because it was located deep in an intronic region. This variant, however, is classified as pathogenic in the germline and likely contributed to the development of this patient’s pancreatic cancer. Not only was the identification of this PV potentially impactful for proper cascade testing, but it also allowed the patient to be considered for a targeted clinical trial.

Another explanation for a germline PV being missed by a somatic lab is due to differences in the interpretation of pathogenicity. Because these tests are designed for different purposes, it is not unexpected that these labs do not have complete concordance regarding the definition of variant pathogenicity. In the case of the *MSH6* variant identified in our report, it was listed as a variant of unknown significance (VUS) and not found on the primary conclusion page. This creates a barrier to easy identification and the necessary database cross-referencing (to prove pathogenic in the germline) for the patients’ providers. The *CHEK2* p.T367fs*15 (c.1100delC) variant identified in this report was identified in 2017 and was listed as a VUS by the somatic lab. While this same lab now classifies it as a PV, somatic labs do not routinely provide reclassification notices that are customary for most germline labs.

Understanding and calculating the scope of germline PVs missed is markedly more difficult. We found that 21 of 320 (6.6%) germline PVs were missed by somatic tumor testing. This may be an underestimation of the true incidence as not all patients seen in our program had germline testing. To the best of our knowledge, this report includes data derived from the largest number of both germline and somatic laboratories, mimicking diverse, real-world experiences. Pauley et al. reported that 20 of 109 (18.3%) germline variants would have been missed by tumor genomic sequencing that was completed by two different laboratories. Lincoln et al. reported that 50 of 617 (8.1%) germline variants identified at a single laboratory were missed by tumor genomic sequencing. In contradistinction to the prior reports, we chose not to include missing genes on the tumor test since this is highly variable and evolving. For example, at least four PVs described by Lincoln et al. were missed by the somatic tumor testing but the tumor test analyzed only 1–2 genes. If we exclude genes missed on the tumor test in those reports, the variants missed by tumor genomic sequencing would have been 16 of 109 (14.7%) and 37 of 617 (6.0%), respectively. These percentages are similar to the data reported here with the implementation of more comprehensive tumor genomic sequencing. In total, these data demonstrate clinically relevant numbers of missed germline PVs.

There are several limitations to our study. First, not all patients underwent germline testing so the true incidence of missed germline PVs could not be calculated. However, our study reflects a real-world practice that takes into account a patient’s personal and family history as well as the financial constraints of germline testing. Further, as would be expected with any tumor testing, there were varying levels of tumor purity which could impact the somatic lab’s ability to detect certain germline alterations. Additionally, we were not able to comprehensively quantify the downstream benefits of identifying germline PV carriers. Finally, variant pathogenicity is inherently dynamic so these results could change overtime.

Both somatic and germline genomic testing play a vital role in improving outcomes for patients with cancer. It is crucial to remember that somatic testing is designed to identify drug targets and is not designed to detect germline PVs. Somatic and germline tests can be complementary to one another with germline tests identifying PVs that can unlock targeted therapy options, and somatic tests uncovering potentially incidental germline PVs. These data, and others, however, reinforce that these tests cannot be used as a substitute for the other. Herein we have highlighted the frequency of germline PVs being missed by somatic testing. These data reinforce the importance of germline testing in patients when clinically indicated, regardless of whether or not a suspicious variant is detected through somatic testing. Appropriate use and implementation of both somatic and germline testing have the potential to improve outcomes for the patient and family members.

### Reporting summary

Further information on research design is available in the Nature Research Reporting Summary linked to this article.

### Supplementary information

REPORTING SUMMARY

## Data Availability

The datasets generated during and/or analyzed during the current study are available from the corresponding author on reasonable request and upon IRB-approval and a data usage agreement.
